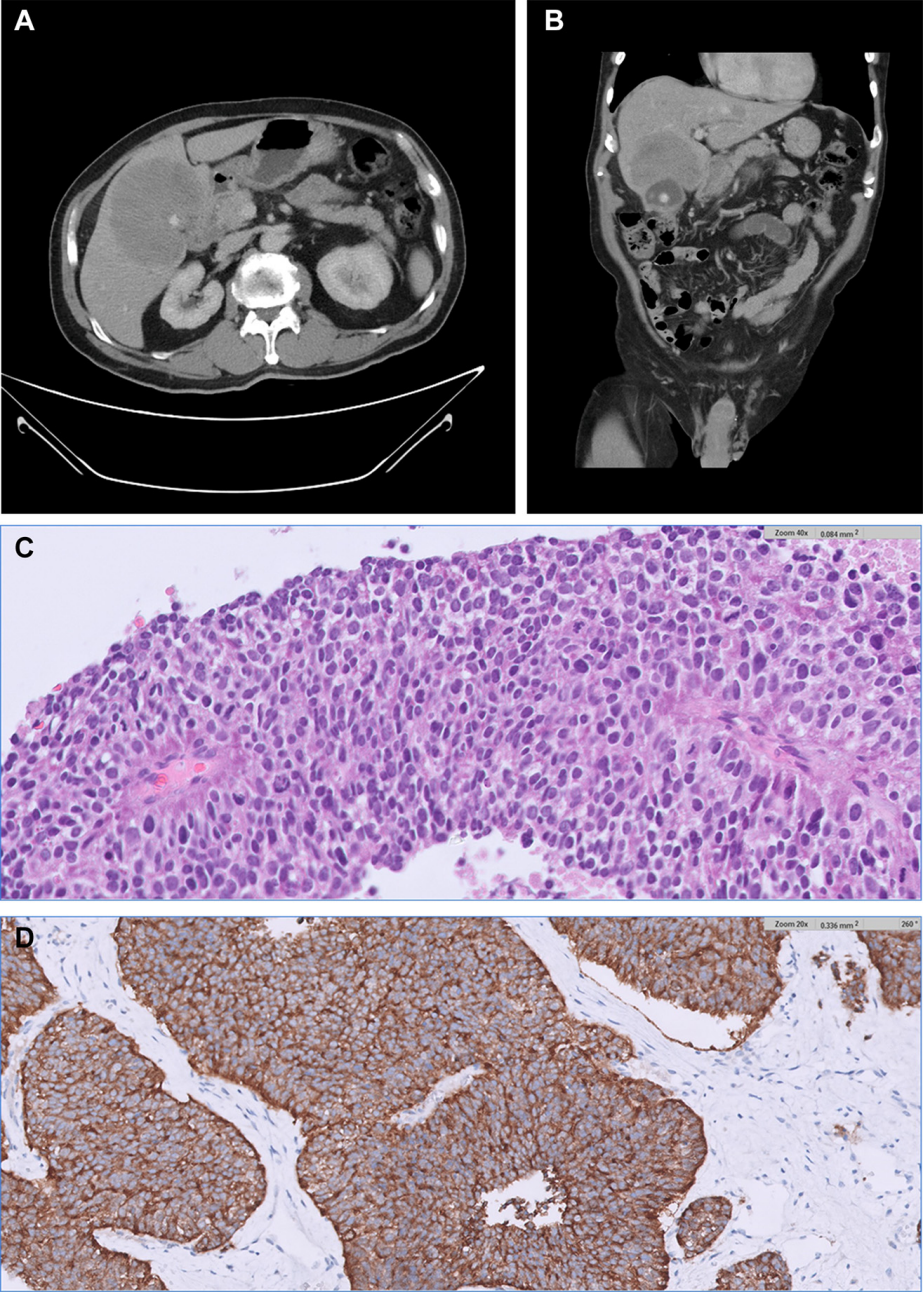# Liver Tumor Encapsulating the Gallbladder

**DOI:** 10.1016/j.gastha.2022.07.011

**Published:** 2022-07-19

**Authors:** Chia-Chu Fu, Nai-Chi Chiu, Chien-Wei Su

**Affiliations:** 1 Division of Gastroenterology and Hepatology, Department of Medicine, Taipei Veterans General Hospital, Taipei, Taiwan; 2 Department of Radiology, Taipei Veterans General Hospital School of Medicine, National Yang-Ming Chiao-Tung University, Taipei, Taiwan; 3 Department of Internal Medicine, School of Medicine, College of Medicine, National Yang Ming Chiao Tung University, Taipei, Taiwan; 4 Hospitalist Ward, Department of Medicine, Taipei Veterans General Hospital, Taipei, Taiwan; 5 Biomedical Science and Engineering Center, National Tsing Hua University, Hsinchu, Taiwan

A 68-year-old man presented with abdominal fullness and icterus. Laboratory data showed hyperbilirubinemia (total bilirubin: 8.2 mg/dL) and elevated liver enzyme (alanine aminotransferase: 607 U/L). Tumor markers revealed only mild elevation of carbohydrate antigen (CA-199: 107 U/mL). Computed tomography scan indicated a well-defined mass at the gallbladder fossa and multiple hypodense lesions in both lobe of liver (Figure A and Figure B). Liver biopsy was arranged. The pathology report revealed medium-size round cells with hyperchromatic and pleomorphic nuclei (Figure C). Immunostaining was positive for synaptophysin (Figure D). The morphological features were compatible with metastatic neuroendocrine neoplasm. For primary origin survey, esophagogastroduodenoscopy, colonoscopy, chest computed tomography scan, and small intestine barium studies were arranged but showed no remarkable finding, suspected primary hepatic neuroendocrine neoplasm. The patient received concurrent chemoradiotherapy with cisplatin and etoposide, and consequently, regressive disease was found after treatment.

This case highlights unusual primary hepatic neoplasms should be considered, despite strong image evidence of common hepatic tumors. Primary hepatic neuroendocrine carcinomas are extremely rare. Primary hepatic neuroendocrine carcinomas are often discovered at older age because of mass effects of the tumor, including abdominal fullness or jaundice. No treatment guidelines were established. In patients with unresectable disease, chemotherapy agents such as 5-fluorouracil, Adriamycin, etoposide, and cisplatin may be useful.

**Figure:**